# Exploring the Antiviral Potential of Tungsten Oxide Nanoparticles Against Herpes Simplex Virus Type 1: A Promising Alternative to Acyclovir

**DOI:** 10.1049/nbt2/6697780

**Published:** 2026-01-06

**Authors:** Abdulhussain Kadhim Jwaziri, Pegah Khales, Seyed Jalal Kiani, Homayoun Yaghouti, Roghayeh Babaei, Zahra Salavatiha, Ahmad Tavakoli

**Affiliations:** ^1^ Department of Microbiology, College of Medicine, University of Kerbala, Karbala, Iraq, uokerbala.edu.iq; ^2^ Department of Virology, School of Medicine, Shiraz University of Medical Sciences, Shiraz, Iran, sums.ac.ir; ^3^ Department of Virology, School of Medicine, Iran University of Medical Sciences, Tehran, Iran, iums.ac.ir; ^4^ Department of Internal Medicine, School of Medicine, Iran University of Medical Sciences, Tehran, Iran, iums.ac.ir; ^5^ Department of Medical Nanotechnology, TeMS.C., Islamic Azad University, Tehran, Iran, ctb.iau.ir; ^6^ Biological Products and Blood Safety Research Center, High Institute for Research and Education in Transfusion Medicine, Tehran, Iran, tmi.ac.ir; ^7^ Research Center of Pediatric Infectious Diseases, Institute of Immunology and Infectious Diseases, Iran University of Medical Sciences, Tehran, Iran, iums.ac.ir

**Keywords:** antiviral activity, herpes simplex virus type 1, HSV-1, nanoparticles, tungsten oxide, WO_3_

## Abstract

Herpes simplex virus type 1 (HSV‐1) is responsible for the majority of cold sores, herpetic keratitis‐induced blindness, profound skin lesions, and encephalitis that can be fatal. Currently, acyclovir and its derivatives are the first‐line therapy for the treatment of HSV‐1 infection. But there are drawbacks to these treatments: limited efficacy against drug‐resistant strains of the virus. Hence, it is of critical importance to explore and develop new antiviral drugs for HSV‐1. In the present study, we explored whether tungsten oxide nanoparticles (WO_3_NPs) were potent inhibitors of HSV‐1 infection as a new class of agent. WO_3_NPs were characterized by X‐ray diffraction (XRD), field‐emission scanning electron microscopy (FE‐SEM), Fourier transform infrared (FTIR) spectroscopy, and zeta potential analysis. Cytotoxicity of Vero cells caused by WO_3_NPs was determined by methyl thiazolyl tetrazolium (MTT) assay. The quantitative real‐time polymerase chain reaction (qRT‐PCR) assay was utilized for further verification of the action of the WO_3_NPs on HSV‐1. The cytotoxicity test showed low toxicity (<20%) of the rod‐shaped WO_3_NPs when they were assayed on Vero cells at concentrations of up to 700 μg/mL. When HSV‐1 was treated with WO_3_NPs at 700 µg/mL [20% cytotoxicity concentration (CC_20_); the concentration causing 20% cytotoxicity, ~80% cell viability] and 1000 µg/mL [50% cytotoxicity concentration (CC_50_); the concentration causing 50% cytotoxicity, ~50% cell viability] for 3 h, the viral load was significantly reduced, achieving inhibition rates of 99.4% and 99.9%, respectively. Additionally, experiments conducted after HSV‐1 infection of Vero cells (post‐treatment assays) indicated that WO_3_NPs at concentrations of 250, 500, and 750 µg/mL significantly suppressed viral replication, with inhibition rates of 82%, 87.5%, and 96.5%, respectively. WO_3_NPs have potent inhibitory effects on HSV‐1. Therefore, they can be considered potential candidates for therapeutic development against infections caused by this virus.

## 1. Introduction

Herpes simplex virus type 1 (HSV‐1) is a member of the genus *Simplexvirus*, the family *Herpesviridae*, the order *Herpesvirales*, the subfamily *Alphaherpesvirinae*, and the species *Human alphaherpesvirus 1* [1]. It is estimated that around 3.8 billion people worldwide, aged under 50 years, representing 64% of this demographic, are affected by HSV‐1 infection [2]. HSV‐1 primarily induces orolabial lesions, although it can also result in genital herpes. Also, HSV‐1 can cause serious diseases like herpetic keratitis or encephalitis [3].

General advances were also achieved in terms of explaining the pathophysiological characteristics and replication methods of HSV‐1, consequently contributing to the production of antiviral medications specifically created for virus elimination [4]. Currently, the drugs of choice for the management of HSV‐1 infections continue to remain acyclovir, valacyclovir, and famciclovir, but other drugs such as trifluridine (exclusively for ophthalmic use), ganciclovir, foscarnet, and cidofovir can also be utilized, with vidarabine being mostly obsolete. All of these drugs, except foscarnet, are incorporated into the developing viral DNA chain and inhibit viral replication as nucleoside analogs. Foscarnet is a pyrophosphate that inhibits viral DNA polymerase [5].

Based on the chronic, recurrent, and untreatable character of HSV‐1 infection, excessive administration of nucleoside analogs, including acyclovir, may induce dramatic side effects, such as neurotoxicity, renal insufficiency, and drug resistance. The development of drug‐resistant HSV‐1 mutants is correlated with reduced drug responses and limited therapeutic options, with devastating effects including long‐standing orolabial lesions, herpetic keratitis, or encephalitis [6]. This highlights the necessity of developing new anti‐HSV‐1 drugs that can overcome these hurdles.

Nanoparticles (NPs) find widespread applications in industrial applications as well as in medicinal applications. Of all the metal NPs, more emphasis is placed on tungsten oxideNPs (WO_3_NPs) because of their unique properties, that is, large surface area and high‐temperature stability [7]. Tungsten (VI) oxide (WO_3_) is a chemical substance of the transition element tungsten with oxygen. It is also prepared as an intermediate when tungsten is prepared from the ores of tungsten [8]. Additionally, bioproducts also make use of WO_3_NPs in the form of pigments, additives, as well as analytical agents [9].

So far, just a few studies have looked at how WO_3_NPs work against certain bacteria, and no studies have looked at how these NPs affect human viruses. Because HSV‐1 is so important and treating infections caused by this virus is so hard, this study aims to see if WO_3_NPs can work as anti‐HSV‐1 medications instead of the ones that are now available.

## 2. Materials and Methods

### 2.1. Characterization of WO_3_NPs

US Research Nanomaterials Inc. provided high‐purity WO_3_ nanopowders (>99%) for this research study. Characterization methods were performed to characterize the NPs using a variety of advanced equipment. X‐ray diffraction (XRD, Philips PW1730) was performed to determine and confirm the crystal structure. Functional groups were identified on the surface using Fourier transform infrared (FTIR) spectroscopy (Thermo Scientific Nicolet Avatar 380). Zeta potential analysis (Horiba SZ‐100) measured the surface charge. The surface morphology and particle size of WO_3_NPs were examined by field‐emission scanning electron microscopy (FE‐SEM, TESCAN MIRA4) operated at an accelerating voltage of 15 kV after sputter coating with a conductive layer.

### 2.2. Cell and Virus

Vero cells, originating from the kidneys of African green monkeys, were cultured in Dulbecco’s modified Eagle’s medium (DMEM; Gibco, Invitrogen, USA), containing high glucose content, and supplemented with 10% heat‐inactivated fetal bovine serum (FBS) (Gibco, Invitrogen, USA), 2 mM L‐glutamine (Merck, Germany) and an antibiotic mixture (100 μg/mL streptomycin and 100 U/mL penicillin; Sigma–Aldrich, USA). The cells were incubated at 37°C in a 5% CO_2_ environment. To determine the antiviral activity, the viral strain HSV‐1 KOS was expanded in Vero cells, and the viral titer of the stock was determined using the Reed and Muench method, reported as TCID_50_/mL. The viral stock was prepared and divided into sterile microtubes for appropriate storage at −80°C for future use [10].

### 2.3. Assessment of Cell Cytotoxicity

The cytotoxic effects of WO_3_NPs on Vero cells were assessed via the methyl thiazolyl tetrazolium (MTT) assay. First, Vero cells were plated at a density of 1 × 10^4^ cells per well in a 96‐well flat‐bottom microtiter plate (SPL Life Science, South Korea) and incubated for 24 h at 37°C. Next, a variety of concentrations of WO_3_NPs (100–1000 μg/mL) were placed in wells in triplicate. The plate was subsequently incubated for 48 h at 37°C. After the incubation period, 10 μL of MTT reagent (5 mg/mL) was added to each well and placed in the dark, 37°C, for 3 h. The MTT solution was then removed carefully, and 50 μL of pure dimethyl sulfoxide (DMSO) (Bio‐Idea, Iran) was added to dissolve the formazan crystals. The plate was gently agitated for 10 min at room temperature. Finally, absorbance was measured at 550 nm using a microplate reader (Hiperion MPR 4+, Roedermark, Germany). Cell viability percentages were calculated by comparing the treated wells to the untreated control group [10].

### 2.4. Determination of Antiviral Activity

#### 2.4.1. Virucidal Assay

To summarize, 100 μL of WO_3_NPs at CC_50_ (the concentration causing 50% cytotoxicity, ~50% cell viability) and at CC_20_ (the concentration causing 20% cytotoxicity, ~80% cell viability) were combined with 100 μL of a HSV‐1 suspension (100 TCID_50_/mL) and incubated for either one or 3 h at 37°C in a humidified atmosphere of 5% CO_2_. A virus control was made with a solution of the virus incubated with a cell culture medium. Mixtures were then applied to Vero cell monolayers and incubated for an hour at 37°C. After incubation, the supernatant was collected, and the cells were washed twice with phosphate‐buffered saline (PBS) to remove any unbound viruses. After washing, the cells had fresh DMEM medium (with 2% FBS) added and were incubated at 37°C for 48 h [10].

#### 2.4.2. Cell Post‐Treatment Assay

Monolayers of Vero cells were cultured and subsequently infected with HSV‐1 solution (100 TCID_50_/mL) at 37°C for 1 h in a 96‐well microtiter plate. Following the HSV‐1 infection, the cells were then washed with PBS in order to eliminate all noninternalized viruses. The infected Vero cells were then treated with factorial concentrations of WO_3_NPs that were considered nontoxic and cultured for 48 h at 37°C and 5% CO_2_. The experiment was further extended to cell controls and virus controls in the same medium, and the viral load of HSV‐1 was determined by applying the quantitative real‐time polymerase chain reaction (qRT‐PCR) technique [10]. The whole assays were carried out in darkness in order to rule out the contribution of the photoactivity of the WO_3_NPs.

### 2.5. qRT‐PCR

The impact of WO_3_NPs on HSV‐1 infection on Vero cells was analyzed via qRT‐PCR. Using the BehPrep Viral Nucleic Acid Extraction Kit (BehGene Biotechnology, Iran), viral DNA was extracted from the supernatants of infected Vero cells (gathered during virucidal and post‐treatment experiments) in accordance with the manufacturer’s instructions. Using the following primers and probe, the qRT‐PCR produced a 75 bp amplicon that targeted the HSV‐1 UL30 gene: forward (5′‐ATCGGCGAGTACTGCATACA‐3′), reverse (5′‐GAGCTCCAGATGGGGCAA‐3′), and probe (5′‐HEX‐ATTCCCTGCTGGTGGGCCA‐BHQ1‐3′). Each 25 μL reaction mixture contained 12.5 μL of RealQ Plus 2x Master Mix for Probe (Ampliqon, Denmark), 2 μL of forward primer (10 µM), 1 μL of reverse primer (10 µM), 1 μL of probe (10 µM), 5 μL of template DNA, and 3.5 μL of ddH_2_O. A Rotor‐Gene Q system (Qiagen, Germany) was used for the amplification process. There were 40 cycles of 95°C for 10 s and 60°C for 30 s after initial denaturation at 95°C for 10 min [10].

The target viral sequence was cloned into the pUC57 vector to generate a reference standard. To create a standard curve, serial tenfold dilutions of the synthesized plasmid, ranging from 10^−1^ to 10^−10^, were made and examined with the test samples. An online DNA copy number calculator (Technology Networks) was used to determine the plasmid copy number. Three copies/μL was the detection limit (LOD) of the qRT‐PCR experiment. Positive and negative controls were included in every PCR run, and all reactions were carried out in triplicate to guarantee reproducibility [10].

### 2.6. Statistical Analysis

Statistical analyses were performed in SPSS software Version 22.0 (IBM, Chicago, IL, USA). The normality of the data was assessed using the Kolmogorov–Smirnov test. The independent *t*‐test was used to assess meaningful differences among quantitative data that followed a normal distribution, and the Mann–Whitney *U* test was used for data that did not follow a normal distribution. Homogeneity of variances was assessed using Levene’s test. A significance threshold of 0.05 was determined to signify statistical significance, where a *p*‐value less than or equal to this level was considered significant. All graphical representation of data was conducted using GraphPad Prism 8.

## 3. Results

### 3.1. Characterization of WO_3_NPs

#### 3.1.1. XRD Analysis

**Figure 1:**
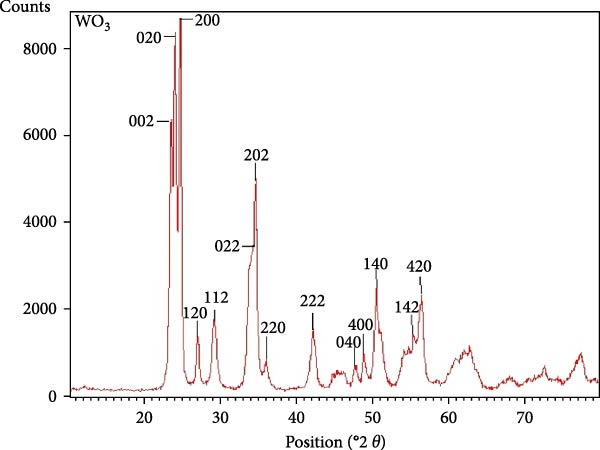
XRD pattern of WO_3_NPs.

The XRD analysis of WO_3_NPs was performed using Cu‐K*α* radiation (*λ* = 1.5406 Å) at 2*θ* values. The diffraction patterns and related planes were interpreted using the Profex 5.4.1 software, which revealed distinct and intense diffraction peaks at 23.15°, 23.67°, and 24.36°, corresponding to the Miller indices (002), (020), and (200), respectively. These peaks confirm the monoclinic phase of the crystal structure of WO_3_NPs. As shown in Figure 1, additional diffraction peaks further validate the monoclinic structure of the WO_3_NPs. This is aligned with the Joint Committee on Powder Diffraction Standards (JCPDS No. 43‐1035), confirming that the synthesized material exists in a single phase [11–14]. The absence of impurity‐related peaks indicates the high purity of the synthesized NP. The results also show that the (200) plane has significantly higher intensity compared to other planes, which may suggest a *possible preferential orientation* along this direction; however, further texture analysis would be required to confirm such growth behavior. The average crystallite size according to the Scherrer equation (*D* = 0.9*λ*/B cos*θ*) was estimated to be ~27.44 nm.

#### 3.1.2. FTIR Analysis

**Figure 2:**
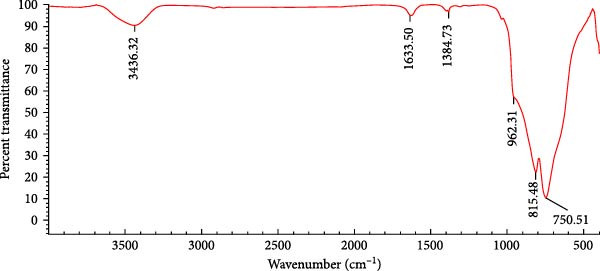
FTIR pattern of WO_3_NPs.

The FTIR spectrum of WO_3_ is shown in Figure 2. The peaks at 3436.32 cm^−1^ and 1633.50 cm^−1^ correspond to O–H stretching vibrations and H–O–H bending vibrations, respectively, indicating the presence of water molecules adsorbed on the NP surface or hydroxyl groups. The weak peak at 1384.73 cm^−1^ can be attributed to impurities or organic residues, such as the symmetric bending vibration of CH_3_ groups, which may be associated with stabilizing agents or precursors used during synthesis. The strong absorption bands observed at 815.84 cm^−1^ and 750.51 cm^−1^ are assigned to the bending vibrations of bridging W–O–W bonds, while the prominent band at 962.31 cm^−1^ corresponds to the stretching vibration of terminal W = O bonds. These assignments are consistent with previous reports on tungsten trioxide [15, 16], which confirm that such vibrational features are characteristic of monoclinic WO_3_ and reflect the complex bonding interactions between tungsten and oxygen atoms.

#### 3.1.3. Zeta Potential

**Figure 3:**
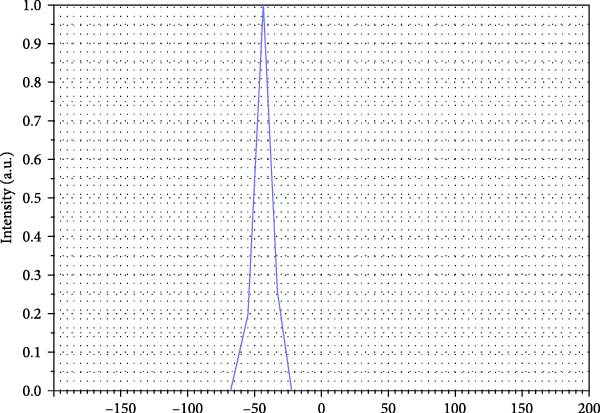
Zeta potential pattern of WO_3_NPs.

For a stable colloidal system to be considered, zeta potential values are usually greater than ±30 mV (whether positive or negative). This threshold ensures sufficient electrostatic repulsion between particles and effectively prevents aggregation [17]. In this study, the WO_3_NPs were dispersed in PBS at neutral pH (~7) with an ionic strength of ~1–10 mM, and measurements were performed at room temperature (~25°C). The zeta potential was measured three times, yielding a mean value of −43.7 ± 0.44 mV, indicating a stable colloidal suspension. The NPs carry a significant negative charge on their surface, creating a repulsive force that prevents their aggregation and contributes to the stability of the system (Figure 3).

#### 3.1.4. FE‐SEM Analysis

**Figure 4:**
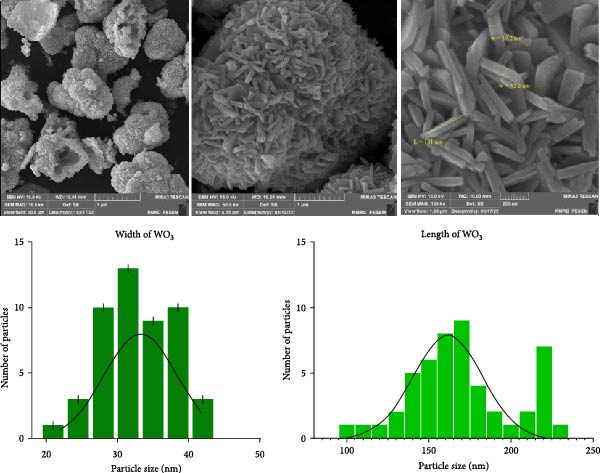
Morphology and size distribution of WO_3_NPs. FE‐SEM image showing elongated rod‐like morphology. Size‐distribution histograms of length and width with Gaussian fits generated using GraphPad Prism 10. A total of 50 nanoparticles were analyzed.

The FE‐SEM analysis of WO_3_NPs revealed an elongated rod‐like morphology. The length and width of a total of 50 NPs were measured separately using FIJI (ImageJ) software. The average width and length of WO_3_NPs were 33.3 ± 5.0 nm and 169.8 ± 31.1 nm, respectively. The relatively larger standard deviation observed in the NP length compared to the width is attributed to the natural variation in rod elongation during the synthesis process, which is commonly observed in rod‐like NP growth. Size‐distribution histograms and Gaussian fits generated using GraphPad Prism 10 are presented in Figure 4, clearly illustrating the variation and distribution of NP dimensions. The uniformity in shape and size distribution suggests well‐controlled synthesis conditions, leading to homogeneous structures. Slight aggregation was observed, likely due to the high surface activity and surface energy of the NPs, as commonly seen in nanorod systems. The results are consistent with XRD analysis, which confirms the presence of characteristic peaks corresponding to the monoclinic crystalline phase of WO_3_. The crystallite size estimated from XRD (~27.44 nm) corresponds to the size of individual crystalline domains, while the larger dimensions observed in FE‐SEM represent the entire rod‐like NPs composed of multiple crystallites. This agreement between FE‐SEM and XRD validates both the synthesis process and the structural characteristics of the NPs. FE‐SEM images were acquired at an accelerating voltage of 15 kV, with a thin gold coating applied to the sample, at magnifications of 10, 50, and 150 Kx, ensuring clear visualization of NP morphology.

### 3.2. Cytotoxicity Assay

**Figure 5:**
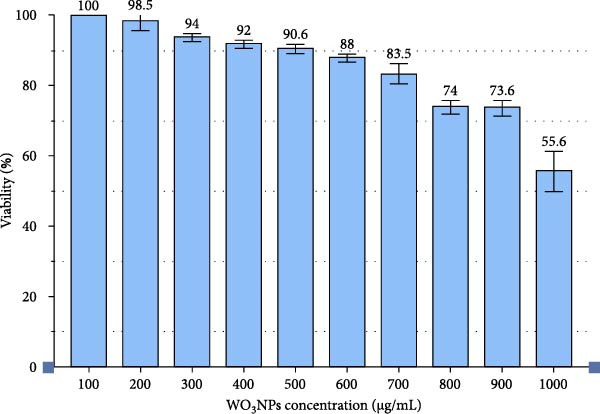
Cytotoxicity of WO_3_NPs on Vero cells. Cell viability was assessed using the MTT assay (performed in triplicate).

The MTT assay results showed that cell viability decreased to 80% (CC_20_, the concentration causing 20% cytotoxicity) and 50% (CC_50_, the concentration causing 50% cytotoxicity) in comparison to control cells when WO_3_NP concentrations increased to 700 and 1000 μg/mL, respectively (Figure 5). Thus, at CC_20_ (700 µg/mL) and CC_50_ (1000 µg/mL) concentrations, we investigated the virucidal potency of WO_3_NPs against HSV‐1. In the post‐treatment assay, the ability of WO_3_NPs to inhibit HSV‐1 replication in Vero cells was tested at nontoxic concentrations up to and including CC_20_ (700 µg/mL).

### 3.3. Antiviral Assays

#### 3.3.1. Virucidal Assay

**Figure 6:**
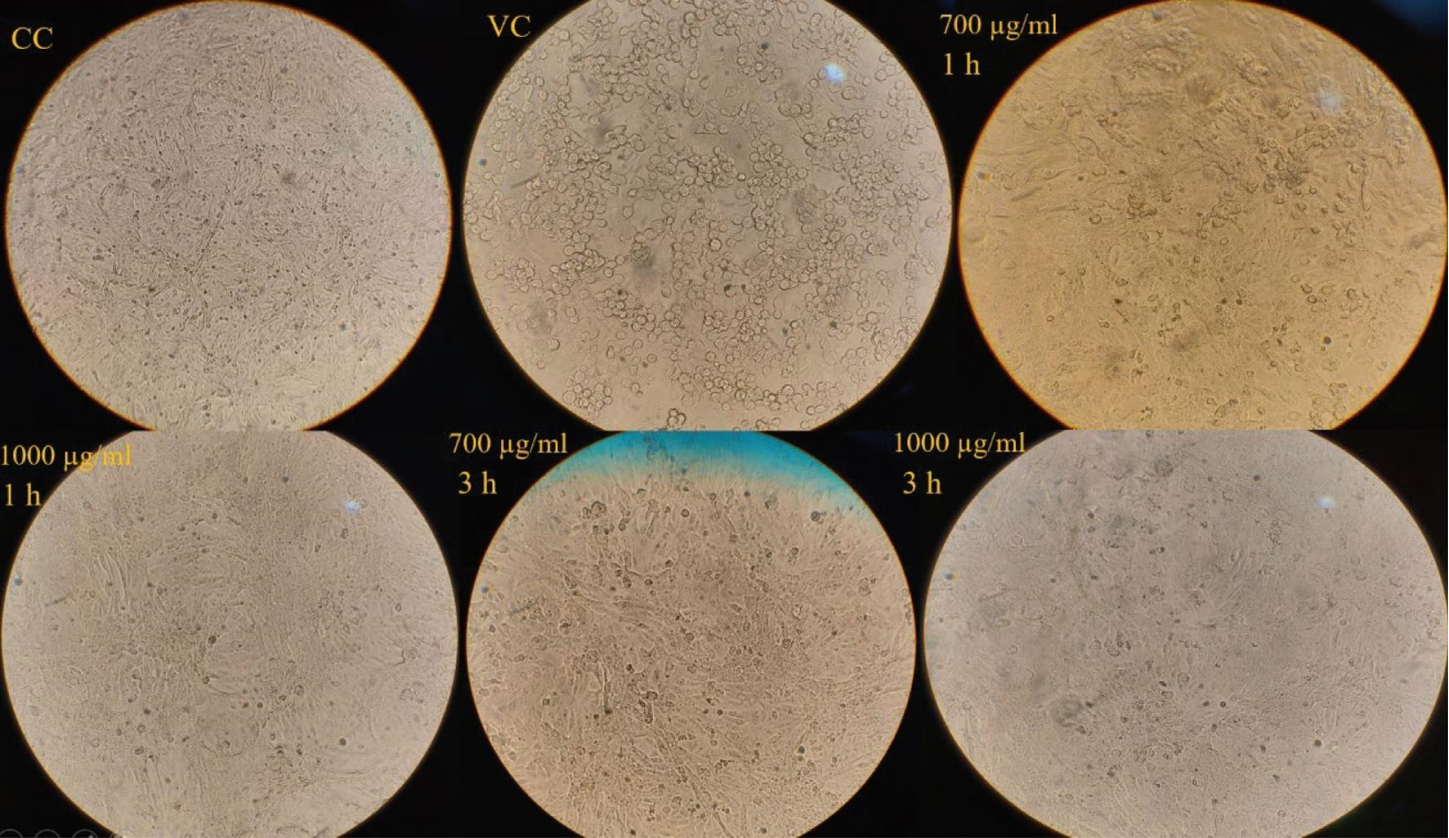
Inhibition of HSV‐1‐induced CPE in Vero cells by 1 h and 3 h incubation of HSV‐1 with WO_3_NPs at different concentrations in the virucidal assay. Visual observation under an inverted microscope (performed in triplicate). CC, cell control; VC, virus control.

The virucidal assay results demonstrated that exposing HSV‐1 to WO_3_NPs for durations of 1 h and 3 h led to a substantial decrease in virus‐induced cytopathic effect (CPE). Furthermore, the CPE inhibition rates were almost the same at both 700 and 1000 µg/mL concentrations (Figure 6).

The qRT‐PCR assay results revealed that elevating the concentration from 700 to 1000 µg/mL caused a decrease in viral load at both 1‐ and 3‐h incubation periods. By utilizing the qRT‐PCR technique for viral load measurement and virus control comparison, the viral inhibitory rates of the WO_3_NPs were determined. Specifically, when the virus HSV‐1 was treated with NPs at the concentration of 700 µg/mL (CC_20_) for 1 h as well as for 3 h, viral suppression degrees were 86.2% (*p* = 0.002) as well as 99.4% (*p* < 0.001), correspondingly. Likewise, when HSV‐1 was treated with higher‐concentration WO_3_NPs of 1000 µg/mL (CC_50_) for time frames of 1 h and also for 3 h, viral inhibition rates were 92% (*p* < 0.001) as well as 99.9% (*p* < 0.001), as revealed in Figure 7.

Figure 7Changes of HSV‐1 viral loads (A) and viral inhibition rates (B) caused by WO_3_NPs in the virucidal assay. (*p* = 0.002 for 700 µg/mL at 1 h, *p* < 0.001 for 700 µg/mL at 3 h, *p* < 0.001 for 1000 µg/mL at 1 h and 3 h, performed in triplicate).  ^∗∗∗^ indicates statistically significant differences (*p* < 0.05). ns indicates not statistically significant (*p* > 0.05). VC, virus control.

**Figure:**
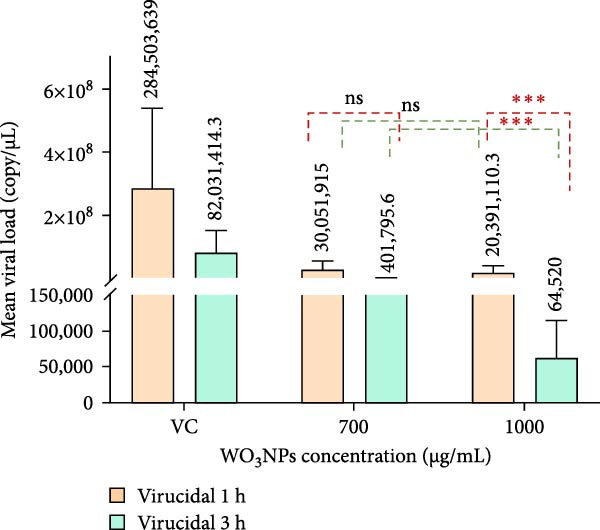
(A)

**Figure:**
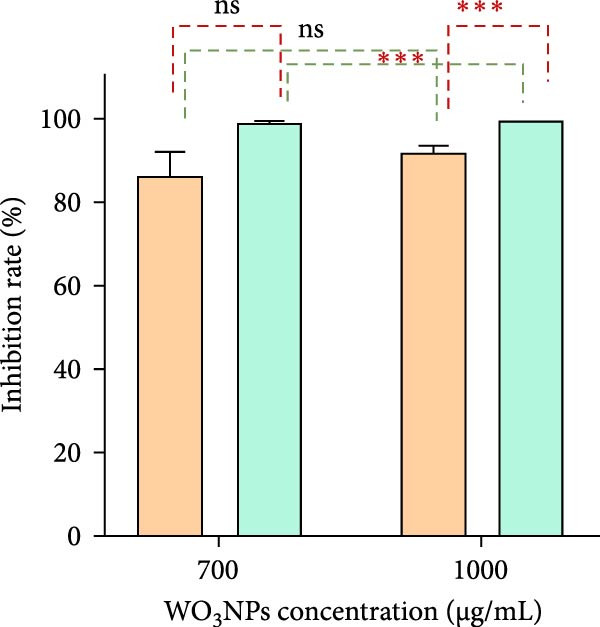
(B)

#### 3.3.2. Post‐Treatment Assay

**Figure 8:**
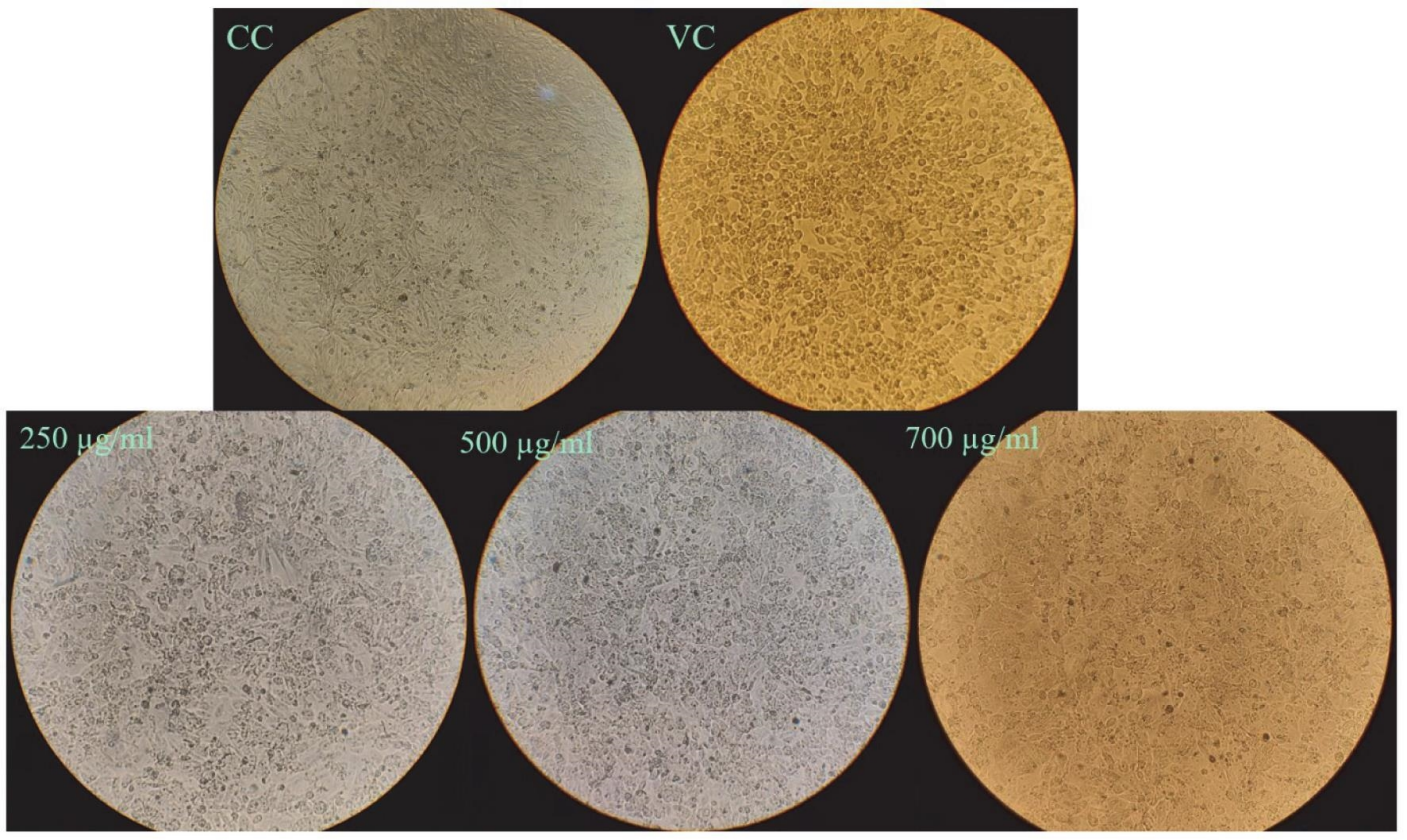
Inhibition of HSV‐1‐induced CPE in Vero cells using WO_3_NPs at different nontoxic concentrations in the posttreatment assay. Visual observation under an inverted microscope (performed in triplicate). CC, cell control; VC, virus control.

The use of subtoxic dosages of WO_3_NPs markedly reduced the CPE generated by HSV‐1 in the infected cells. The most pronounced reduction in CPE formation was observed at the highest concentration tested, 700 μg/mL (Figure 8). Analysis via qRT‐PCR demonstrated that WO_3_NPs at concentrations of 250, 500, and 750 μg/mL significantly decreased the number of HSV‐1 genomic DNA copies, achieving inhibition rates of 82%, 87.5%, and 96.5%, respectively (*p* < 0.001) (Figure 9 and Table 1).

Figure 9Changes of HSV‐1 viral loads (A) and viral inhibition rates (B) caused by WO_3_NPs in the posttreatment assay. (*p* < 0.001 for 250, 500, and 750 µg/mL vs control, performed in triplicate).  ^∗∗∗^ Indicates statistically significant (*p* < 0.05). VC, virus control.

**Figure:**
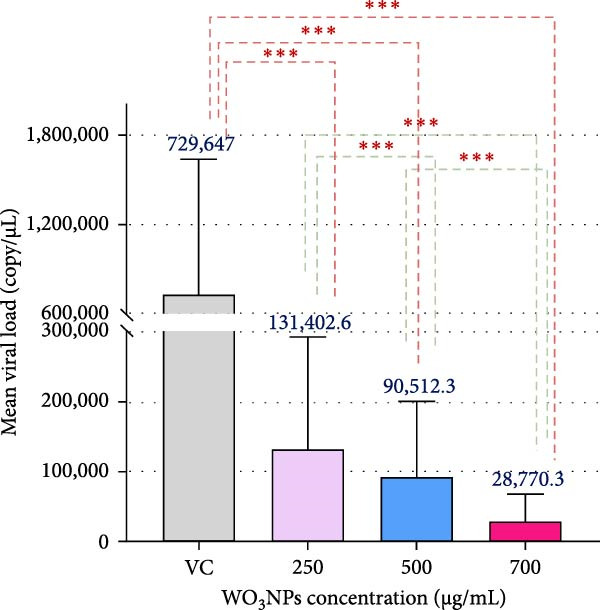
(A)

**Figure:**
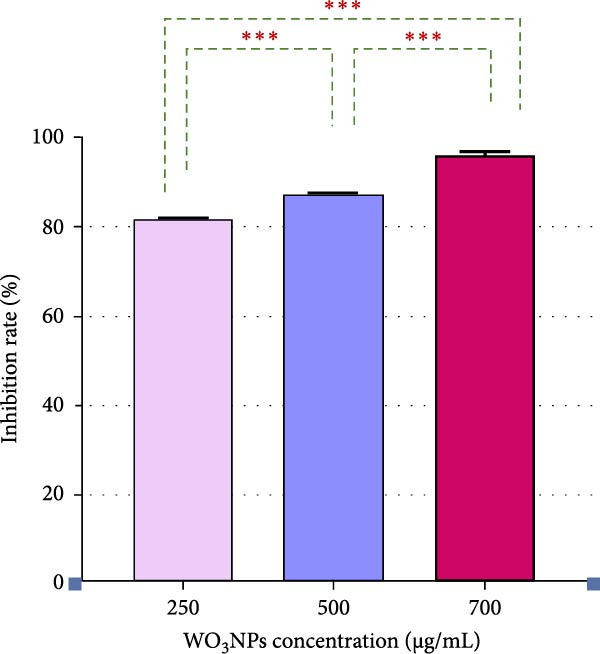
(B)

**Table 1:** Comparison of the antiviral activity between different concentrations of WO_3_NPs against HSV‐1 in the virucidal and post‐treatment assay.

WO_3_NPs	Compared groups	Average difference (%)	Confidence interval (CI, %)	*p*‐Value
Posttreatment	250 µg/mL vs. VC	82	81.8–82.3	<0.001 ^∗^
	250 vs. 500 µg/mL	5.4	5.1–5.7	<0.001 ^∗^
	250 vs. 700 µg/mL	14.4	13.5–15.3	<0.001 ^∗^
	500 µg/mL vs. VC	87.5	87.3–87.6	<0.001 ^∗^
	500 vs. 700 µg/mL	9	7.7–10.2	0.001 ^∗^
	700 µg/mL vs. VC	96.5	95.1–97.8	<0.001 ^∗^

Virucidal (1 h)	700 µg/mL vs. VC	86.2	71.6–100.7	0.002 ^∗^
	700 vs. 1000 µg/mL	5.8	−15.4–3.8	0.169
	1000 µg/mL vs. VC	92	88.2–95.7	<0.001 ^∗^

Virucidal (3 h)	700 µg/mL vs. VC	99.4	99.3–99.6	<0.001 ^∗^
	700 vs. 1000 µg/mL	0.43	0.24–0.61	0.003 ^∗^
	1000 µg/mL vs. VC	99.9	99.7–100	<0.001 ^∗^

## 4. Discussion

According to the findings of the current study, WO_3_NPs showed a virucidal impact that reduced the viral load after exposure to the virus for 1 and 3 h at the two concentrations that were studied (700 and 1000 µg/mL). Furthermore, increasing concentration and exposure time enhanced the extent of viral load reduction. This indicates that the antiviral effects of WO_3_NPs are both dose–dependent and time–dependent. Specifically, as the exposure time and concentration increase, the antiviral effects are significantly enhanced.

The second set of experiments was to determine the efficacy of WO_3_NPs in a post‐infection environment. The aim was to find out if the NPs were capable of inhibiting or interrupting viral replication at some time after the virus had infected the cells. The results of the study indicated that the antiviral efficiency of the WO_3_NPs was enhanced with increased dosages, with the best inhibitory potential being realized in the maximum inhibitory concentration of 700 µg/mL.

As we were already familiar with the photoactivity of WO_3_NPs, such that they create reactive oxygen species (ROS) when illuminated for enhancing their antimicrobial activity [18], it is noteworthy that all of our assays were conducted in the dark in order to prevent such an effect. It thus suggests that the achieved antiviral activity is most likely due to mechanisms that do not involve ROS formation, that is, direct interaction with viral entities or inhibition of the process of cell uptake.

The results of the cytotoxicity assay of MTT also indicated that the WO_3_NPs exhibit biocompatible and safe properties, whereas higher toxicity was exhibited by copper oxide (CuO) and zinc oxide (ZnO) NPs in similar Vero cell models [19, 20]. It may be concluded from the findings that the WO_3_NPs may be placed in the list of safe and biocompatible nanomaterials.

Up to the present, no research study has explored the antiviral behavior of WO_3_ nanostructures against any virus of human or animal origin; thus, the present study is original. Furthermore, few research works have analyzed their effectiveness against bacteria, and more than that, there is still limited research on the antibacterial potential of WO_3_NPs. As an example, the antibacterial potential of WO_3_ nanorods against *Escherichia coli* was investigated by Ghasempour et al. [21] in darkness and in the presence of visible light. Exposing the WO_3_ nanorods to visible light, in fact, showed a strong antibacterial potential with a >92% inactivation of bacteria after exposure for 24 h at room temperature. Baroot et al., in another research, analyzed the antibacterial potential of WO_3_NPs against *Staphylococcus aureus* and *Pseudomonas aeruginosa* [22]. As per data, the WO_3_NPs notably inhibit the action of *P. aeruginosa* and *S. aureus*. Bashir et al. [23] assessed antimicrobial properties of WO_3_NPs coated by antibiotics (ampicillin, penicillin, and ciprofloxacin). According to their results, WO_3_NPs paired with ciprofloxacin demonstrated the highest antibacterial activity, followed by those linked with penicillin and ampicillin. In line with these studies, Matharu et al. [24] evaluated the antimicrobial activity of WO_3_NPs against Gram‐negative and Gram‐positive bacteria. For the first time, this study also investigated the effects of WO_3_NPs on a DNA virus (bacteriophage T4). Based on their findings, WO_3_NP at high concentrations has shown remarkable efficacy against bacteriophage T4 and Gram‐positive bacteria (*S. aureus*), making it an appropriate antimicrobial agent.

However, there are a limited number of studies investigating the antiviral activity of other forms of tungsten NPs. Overall, the discovery of antiviral and antibacterial functions of tungsten has originated from the Ren et al. [25] patent, demonstrating the virucidal efficacy of tungsten NPs, especially combined with other effective antimicrobial components. According to this study, tungsten carbide reduced the avian H5N1 influenza virus by 99.99% after a treatment time of 30 min. Antiviral properties of tungsten carbide NPs against four viruses, vaccinia virus, human adenovirus type 5, poliovirus type 1, and murine norovirus, were also investigated in another study performed by Pfaff et al. [26]. Findings demonstrated that tungsten carbide NPs could decrease the infectivity of all four viruses by at least four log_10_ of TCID_50_/mL after 15 min. Similar to our results, a dose–effect curve demonstrated that the virucidal activity of NPs depended on particle concentration, and their virucidal activity increased with incubation time. According to these results, tungsten carbide NPs hold great promise for developing novel disinfection methods.

The results of our investigation have demonstrated that the antiviral activity of WO_3_NPs in the post‐treatment experiment (after virus adsorption) is enhanced by increasing their concentration. When the virucidal activity of WO_3_NPs was evaluated at various concentrations, a similar increased antiviral activity was observed. The direct impact of WO_3_NPs on viral particles is assessed in the virucidal assay, while the influence of WO_3_NPs on different stages of viral replication is investigated in the post‐treatment analysis.

It should be noted that HSV‐1 replication occurs within host cells. Therefore, the higher the uptake of WO_3_NPs into the cells increases the likelihood of interfering of WO_3_NPs with different stages of the HSV‐1 replication cycle. As electron microscope images show, WO_3_NPs exhibit a rod‐like morphology. Previous observations indicate that the morphologies of NPs affect the mechanisms of endocytosis [27]. While spherical NPs are isotropic, rod‐shaped WO_3_NPs present axial and radial facets, which may enhance their interactions with viral particles or cellular surfaces [27].

In addition to morphology, the size of WO_3_NPs is a key factor contributing to their antiviral activity. In our study, the average size of WO_3_NPs was ~45 nm. The antiviral and antimicrobial activity of NPs is influenced by multiple factors, including their chemical composition, protein corona, and dose metrics (e.g., mass versus surface area), in addition to size [28, 29]. These factors collectively contribute to the high antiviral activity of WO_3_NPs observed in this study.

One of the drawbacks of the current study is that the specific mechanism of WO_3_NPs’ antiviral action has not been determined. Additionally, our study did not assess the impact of WO_3_NPs against HSV‐1 in other conditions, such as pretreatment of cells with WO_3_NPs before viral infection (pretreatment assay) or simultaneous treatment of cells with WO_3_NPs and HSV‐1 (co‐treatment assay).

## 5. Conclusion

This study is the first to investigate the antiviral activity of WO_3_NPs against a human virus. The findings of this investigation have demonstrated that WO_3_NPs can inhibit HSV‐1 via two different mechanisms. In the first mechanism, the virus is directly impacted by these NPs, resulting in virus inactivation. The virus may be deactivated by this mechanism in a number of ways. WO_3_NPs, for instance, can adhere to the virus or its ligands and stop it from binding to its host receptor. This inhibits the virus’s ability to enter cells and stops infection. Furthermore, it is likely that the WO_3_NPs can physically induce structural damage to the virus, resulting in viral inactivation. Meanwhile, findings of the present study indicated that the incorporation of WO_3_NPs following HSV‐1 infection of the cells is capable of lowering viral load. This further suggests that the viral replication process in the life cycle could be obstructed by these NPs at some stage. Ultimately, based on the positive findings of the present study, WO_3_NPs shall remain a potential candidate for further preclinical studies for fighting off HSV‐1 infection. Nevertheless, considering that the present study is restricted to in‐vitro Vero cell cultures and deals solely with the strain of HSV‐1 KOS, the results may not directly extrapolate into in‐vivo settings in humans or in animal models or into other subtypes of HSV‐1. Notable parameters, including immune response, drug delivery, and system toxicity, among others, remain unexplored. Further research employing laboratory animals, such as mouse or rat models, and testing additional HSV‐1 strains, including acyclovir‐resistant strains, is necessary to validate these results and explore their therapeutic potential.

## Conflicts of Interest

The authors declare no conflicts of interest.

## Author Contributions

Ahmad Tavakoli conceptualized, supervised and administrated the study. Abdulhussain Kadhim Jwaziri, Pegah Khales, Homayoun Yaghouti, and Zahra Salavatiha performed all experiments. Seyed Jalal Kiani and Zahra Salavatiha performed statistical analysis. Seyed Jalal Kiani contributed to interpretation of the results. Roghayeh Babaei performed all interpretations related to nanomaterials. Abdulhussain Kadhim Jwaziri and Pegah Khales wrote the original manuscript. Ahmad Tavakoli performed revisions. All authors reviewed and edited the manuscript.

## Funding

This study was financially supported by the Iran University of Medical Sciences (Grant Number: 1401‐2‐99‐23783).

## Data Availability

Data sharing is not applicable to this article as no new data were created or analyzed in this study.
